# Nonlinear Magnetoelectric Response of Planar Ferromagnetic-Piezoelectric Structures to Sub-Millisecond Magnetic Pulses

**DOI:** 10.3390/s121114821

**Published:** 2012-11-02

**Authors:** Florian Kreitmeier, Dmitry V. Chashin, Yury K. Fetisov, Leonid Y. Fetisov, Irene Schulz, Gareth J. Monkman, Mikhail Shamonin

**Affiliations:** 1 Faculty of Electrical Engineering and Information Technology, Regensburg University of Applied Sciences, Seybothstr. 2, 93053 Regensburg, Germany; E-Mails: florian.kreitmeier@gmx.net (F.K.); irene1.schulz@hs-regensburg.de (I.S.); gareth.monkman@hs-regensburg.de (G.J.M.); 2 Faculty of Electronics, Moscow State Technical University of Radio Engineering, Electronics and Automation, Prospekt Vernadskogo 78, 119454 Moscow, Russia; E-Mails: dchashin@list.ru (D.V.C.); fetisov@mirea.ru (Y.K.F.); 3 Faculty of Physics, M.V. Lomonosov Moscow State University, GSP-1, 119991 Moscow, Russia; E-Mail: fetisovl@yandex.ru

**Keywords:** composite materials, magnetoelectric effect, magnetostriction, piezoelectricity, magnetic field pulse

## Abstract

The magnetoelectric response of bi- and symmetric trilayer composite structures to pulsed magnetic fields is experimentally investigated in detail. The structures comprise layers of commercially available piezoelectric (lead zirconate titanate) and magnetostrictive (permendur or nickel) materials. The magnetic-field pulses have the form of a half-wave sine function with duration of 450 μs and amplitudes ranging from 500 Oe to 38 kOe. The time dependence of the resulting voltage is presented and explained by theoretical estimations. Appearance of voltage oscillations with frequencies much larger than the reciprocal pulse length is observed for sufficiently large amplitudes (∼1–10 kOe) of the magnetic-field pulse. The origin of these oscillations is the excitation of bending and planar acoustic oscillations in the structures. Dependencies of the magnetoelectric voltage coefficient on the excitation frequency and the applied magnetic field are calculated by digital signal processing and compared with those obtained by the method of harmonic field modulation. The results are of interest for developing magnetoelectric sensors of pulsed magnetic fields as well as for rapid characterization of magnetoelectric composite structures.

## 1. Introduction

Magnetoelectric (ME) interactions in planar composite structures comprising mechanically coupled ferromagnetic (FM) and piezoelectric (PE) layers have been investigated intensively in recent years due to the prospects of their application as sensitive magnetic field sensors [1–3]. The functional principle of a ME sensor is as follows: when the structure is placed into the magnetic field, the magnetostriction causes a deformation of the FM layer. This strain is passed to the PE layer and a voltage is generated between the electrodes of the PE layer due to the PE effect. This voltage is proportional to the measured field strength. To create sensors for various types of magnetic field, detailed studies of the behavior of ME structures in weak low-frequency harmonic fields, in static magnetic fields and in pulsed magnetic fields are needed.

From the literature it is clear that the behavior of ME structures in harmonic low-frequency magnetic fields has already been intensively investigated. It was shown that the amplitude of the voltage *u* generated by ME structure in a weak harmonic field is proportional to *d × q × δh*, where *d* is the PE coefficient of the PE layer, *q* = ∂λ/∂*H* is the piezomagnetic coefficient, *H* is the magnetic field strength, *λ* is magnetostriction of the FM layer and δ*h* is the amplitude of the alternating magnetic field. The voltage *u* reaches its maximum when a bias magnetic field *H*_m_, at which the coefficient *q* takes its largest value, is applied to the structure. *H*_m_ is usually in the order of few Oe to a few kOe, depending on the properties of the FM layer. The voltage amplitude increases further at 1–2 orders of magnitude when the field frequency coincides with a frequency of acoustic oscillations of the structure due to the resonant enhancement in the PE strain [4]. Feasibility of making sensors for alternating magnetic fields with a detection limit down to ∼10^−10^ T [5] in the frequency range 1 mHz–10 kHz [6,7] employing composite structures with different material compositions and geometries was demonstrated. An equivalent magnetic noise floor of ∼10^−1^–10^1^ pT·Hz^−1/2^ at a resonance frequency of ∼250 Hz was recently achieved using thin-film [8] and lamination [9] technologies. It was also shown that a cross-modulation scheme can shift low-frequency signals to a higher frequency in order to achieve a lower noise floor [10].

When the ME structure is placed into a permanent or slowly varying field *H(t)* with a large amplitude, the magnetostriction causes a quasi-static deformation of the PE layers, the appearance on their electrodes of bound charges *Q* and a corresponding DC voltage. In this case the voltage is proportional to the magnetostriction *u*(*H*)∼*d* × λ(*H*). Initially it grows with increasing *H* and then reaches a constant level at field strengths where the magnetostriction saturates. At the same time, due to the finite conductivity of the PE layers in the structure, a partial compensation of bound charges by free charges takes place, leading to reduction of the resulting voltage across the electrodes of the structure with time [6]. Therefore, the voltage across the electrodes of the FM-PE structure placed into a constant or slowly varying magnetic field of large amplitude depends not only on the field strength, but also on the time of measurement. For this reason, ME structures are less suitable for creating sensors for static or slowly varying magnetic fields.

Only a few experimental studies [11–15] have been devoted to the transient behavior of ME materials and structures in pulsed magnetic fields. For the first time the generation of voltage pulses with amplitudes up to 70 mV has been observed at the electrodes of a single crystal Cr_2_O_3_ when this crystal was magnetized with pulses of duration ∼1 ms and an amplitude of up to 4 kOe [11]. In [12] the generation of an alternating voltage with a frequency equal to the frequency of the planar acoustic oscillations was observed in the ceramic structure consisting of alternating layers of nickel-zinc ferrite and lead zirconate titanate (PZT), when it was exposed to field pulses of small amplitude *Ĥ*∼10–100 Oe with the length of 1–100 μs. Additionally the structure was tangentially magnetized with a bias field *H* ≈ 250 Oe. Note that the pulses were short, *i.e.*, the frequency of the acoustic oscillations was smaller than the reciprocal pulse length. The possibility of applying such a technique combined with the Fourier transform to measurements of the frequency characteristics of ME structures in the frequency range from tens of Hz to several MHz was demonstrated [13]. In [14,15] a sample of a bulk ceramic composite containing a mixture of cobalt ferrite and barium titanate particles was magnetized by pulses of duration 4–50 ms with a peak-to-peak value of up to 50 kOe and the voltage pulses generated by the structure were recorded. According to the measurements obtained dependence of the efficiency of ME interaction on the field reached a maximum of ≈ 5.5 mVOe^−1^cm^−1^ in fields of ≈ 2–5 kOe. Hysteresis between the generated voltage and magnetic field strength has been measured. The authors explained this hysteresis by the strong influence of the charge compensation processes in the PE phase of the composite.

The purpose of this paper is a study of the ME response of planar FM-PE structures to magnetic-field pulses with fixed duration in a sub-millisecond range. The most interesting case when the pulse amplitude exceeds the saturation field of the FM layer and various types of acoustic oscillations in the structures are excited is considered. No bias magnetic field is applied. The pulse length is sufficiently short for neglecting the charge compensation due to the finite conductivity of the PE layer. On the other hand, the magnetic pulse is much longer than the period of observed oscillations. As the object of studies the structures containing layers of commercially available cobalt-iron alloy (CoFe) or nickel (Ni), possessing high magnetostriction and large enough saturation fields, and the layers of PZT ceramics, having large piezoelectric modulus, are selected. The paper starts with the description of the samples and the research methodology. Then the results of measurements together with their discussion and theoretical estimates are presented. The possibility of using the described pulsed technique for rapid characterization of ME interactions in composite structures is demonstrated. In conclusion the main findings of the work are summarized and the recommendations for the use of ME structures for the measurement of pulsed magnetic fields are given. The notation used in the paper is summarized in Appendix Table A1.

## 2. Samples and Measurement Methodology

**Figure 1.**
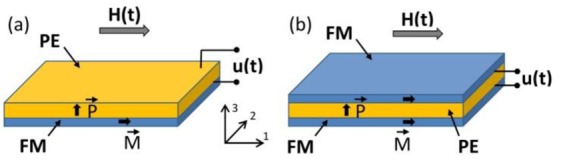
Geometry of bilayer (**a**) and trilayer (**b**) structures.

Measurements were made on bi- and symmetric trilayer structures, containing layers of FM metallic materials (CoFe alloy or Ni) and the piezoceramic layer of PZT: CoFe/PZT, Ni/PZT, CoFe/PZT/CoFe and Ni/PZT/Ni (see Figure 1). The FM layers were made from either CoFe alloy VACOFLUX50^®^ (permendur with the nominal composition Co_0.49_Fe_0.491_V_0.019_, manufacturer Vaccumschmelze GmbH & Co. KG, Hanau, Germany) or Ni (Ni201, manufacturer ATI Allegheny Ludlum, Remscheid, Germany). The PE layers were made from the PE ceramics VIBRIT^®^ 1100 (manufacturer Johnson Matthey Catalysts, Redwitz, Germany). Layers of FM materials and PE ceramics had the following planar dimensions: length *L* = 10 mm, width *b* = 5 mm. The thickness of FM layer *a*_m_ was 0.35 mm for CoFe and 0.5 mm for Ni, the thickness of PZT *a*_p_ = 0.5 mm. The surfaces of the PZT layer were coated with Au electrodes before being poled perpendicularly to its plane. The FM plates and PZT layer were bonded mechanically by an epoxy adhesive. The arrows in Figure 1 show the direction of the magnetization of the FM layer and the direction of the polarization of the PE layer. This is the so-called L-T configuration [16]. The magnetic field was applied in the plane of the samples along the long side of the structures. The voltage pulses *u*(*t*) generated by the structures were measured between the electrodes of the PZT layers.

**Figure 2.**
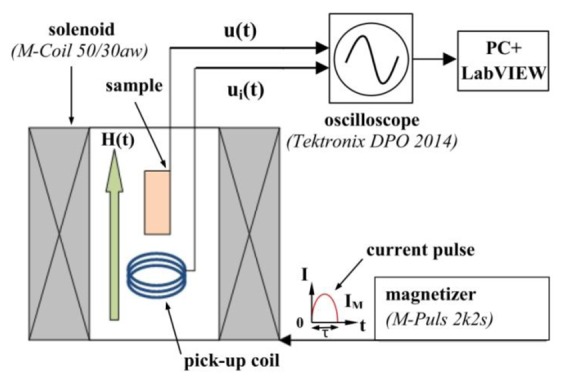
Schematic diagram of the measurement setup.

The block diagram of the measuring apparatus is shown in Figure 2. The sample was placed inside the solenoid of a conventional magnetizer *M-Pulse 2k2s* with *M-Coil 50/30aw* (M-Pulse GmbH & Co. KG, Kierspe, Germany). Magnetic-field pulses *H*(*t*) had the form of a half-wave sine function with a constant duration *τ* = 450 μs and amplitudes between 500 Oe and 38 kOe. In order to measure the shape and the magnitude of the field pulses, the pick-up coil (3 turns of copper wire with a diameter of 0.3 mm) with a diameter of 11 mm was placed inside the solenoid in the vicinity of the sample. Time-dependence of the magnetic field *H*(*t*) was determined by integrating the voltage *u*_i_(*t*) induced in the pick-up coil. The voltage pulses with an amplitude of up to 27 V generated by a PZT layer were recorded using a digital storage oscilloscope *Tektronix* (DPO 2014) with the input resistance of 10 MΩ. The data files contained up to 125 × 10^3^ points.

The frequency spectrum of the voltage pulse *g*(*f*) was calculated using the discrete Fourier transform function in LabView. For all CoFe samples the dependencies of the ME conversion coefficient on the frequency *f* at DC bias field *H*_m_ and at fixed frequency *f* on the DC field *H* were also obtained by the standard method of harmonic field modulation (HFM) (*δh* = 1 Oe, *f* = 100 Hz–300 kHz, bias field *H*_m_ = 650 Oe) [17].

## 3. Results and Discussion

### 3.1. Measurement Results

**Figure 3.**
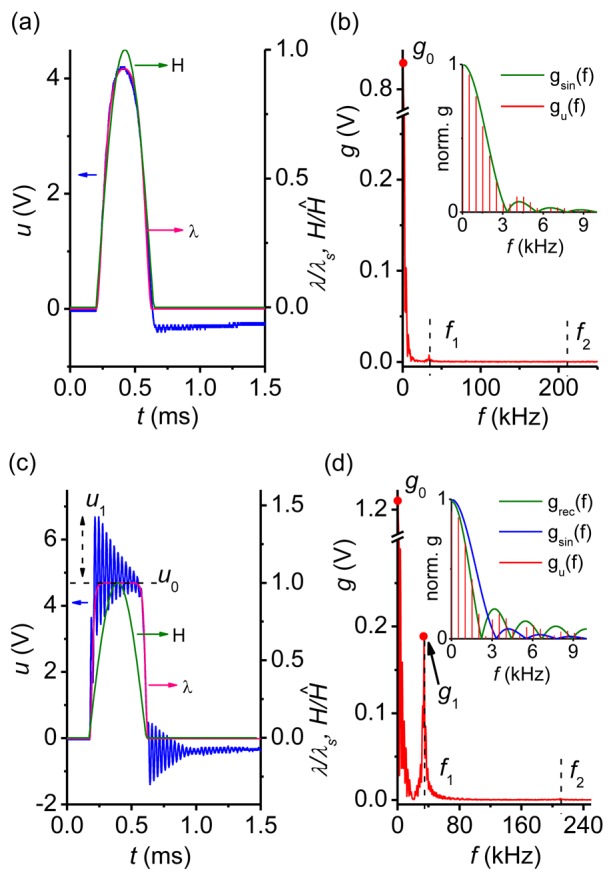

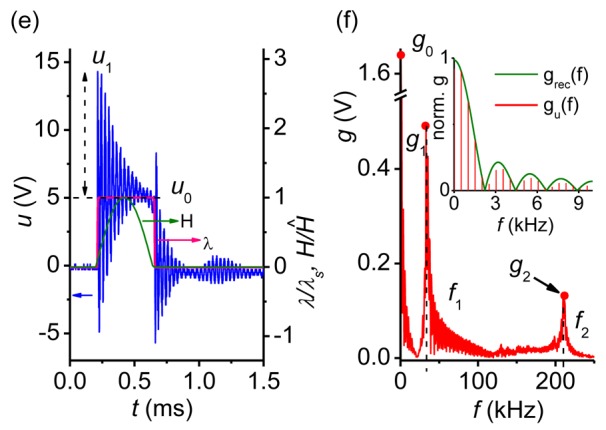
The time dependence *u*(*t*) and the frequency spectra *g*(*f*) of voltage pulses generated by CoFe/PZT structure when excited by magnetic field pulses with different amplitudes *Ĥ*: (**a**) and (**b**) −1.5 kOe, (**c**) and (**d**) −6.5 kOe, (**e**) and (**f**) −38 kOe. Here and in the following *u*_o_ denotes the amplitude of voltage pulse without oscillations. The insets compare the spectrum of the voltage pulse *g*_u_(*f*) with that of a half-sine pulse *g*_sin_(*f*) and of a rectangular pulse *g*_rec_(*f* ) with the pulse length *τ*, with their values normalized at *f* = 0 Hz.

Figure 3 shows the evolution of both the time dependence *u*(*t*) and the frequency spectrum *g*(*f*) of the voltage pulse *u*(*t*) generated by the CoFe/PZT structure dependent on the amplitude *Ĥ* of the exciting magnetic-field pulse *H*(*t*).

With the increase of the amplitude of the field pulse its shape *H*(*t*)/*Ĥ* and frequency spectrum remain practically unchanged. For *Ĥ* < 1.5 kOe the shape of the voltage pulse *u*(*t*) follows the time dependence of the field strength *H*(*t*). When *Ĥ* is increased up to ≈ 1.5 kOe, a voltage pulse with a flat top starts to form (Figure 3(a,b)). The insets in the right column of Figure 3 illustrate how the low-frequency (*f* < 9 kHz) part of the voltage pulse spectrum changes from a half-sine pulse to a rectangular pulse. The amplitude *u*_0_ of the generated pulse reached ≈ 5 V. With further increase of *Ĥ* the damped oscillations of the voltage pulse have been observed on its flat top and immediately after the end of the excitation pulse. These oscillations have the initial amplitude *u*_1_ (Figure 3(c)) and the harmonic frequency *f*_1_ ≈ 33 kHz (Figure 3(d)) in the frequency spectrum. As shown below, these low-frequency oscillations with frequency *f*_1_ correspond to the excitation of flexural acoustic oscillations of the structure. For the excitation pulse amplitudes *Ĥ* ≥ 11 kOe, the high-frequency oscillations of *u*(*t*) with frequency *f*_2_ ≈ 210 kHz and amplitude *u*_2_ (Figure 3(e)) have appeared additionally on the top of the pulse and immediately after excitation which is clearly visible in the spectrum of the signal (Figure 3(f)). These high-frequency oscillations, as it will be shown below, are caused by the excitation of the planar acoustic oscillations in the structure. Acoustic oscillations enhance deformations in the PE layer (electromechanical resonance) leading to voltage values higher than the ME voltage resulting from quasi-static magnetostriction.

**Figure 4.**
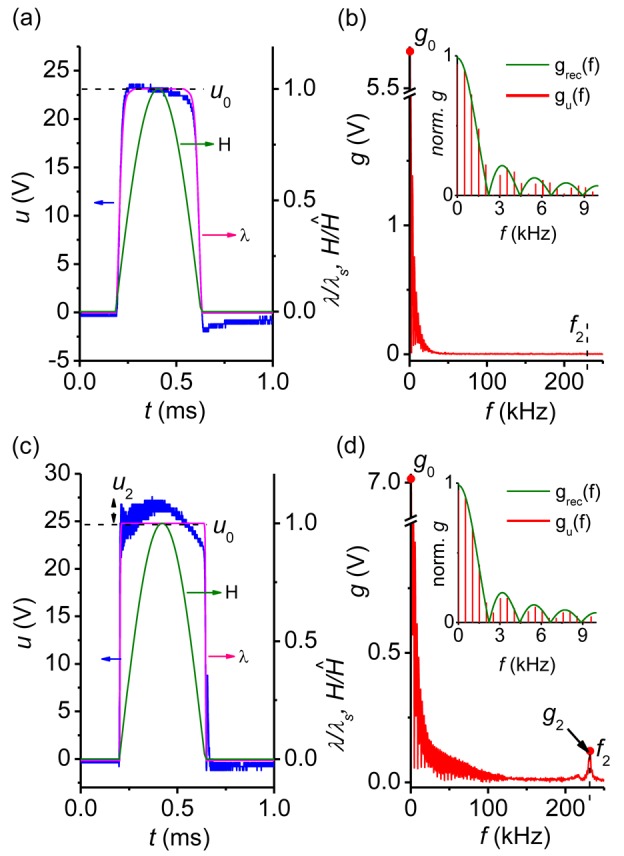
The time dependence *u*(*t*) and the frequency spectra *g*(*f*) of voltage pulses generated by symmetric CoFe/PZT/CoFe structure when excited by magnetic field pulses with different amplitudes *Ĥ*: (**a**) and (**b**) −6.5 kOe, (**c**) and (**d**) −38 kOe. The insets compare the spectrum of voltage pulse *g*_u_(*f*) with that of a rectangular pulse *g*_rec_(*f*), with the pulse length *τ*, with their values normalized at *f* = 0 Hz.

Figure 4 presents the evolution of both the time function *u*(*t*) and the frequency spectrum *g*(*f*) of the voltage pulse with growing amplitude *Ĥ* of the exciting magnetic field *H*(*t)* in the symmetrical trilayer structure CoFe/PZT/CoFe. In this case only a gradual formation of the flat top pulse (Figure 4(a)) with an amplitude of up to *u*_0max_ ≈ 24 V and the broadening of its spectrum occurred with increasing *Ĥ* (Figure 4(b)). The insets in the right column of Figure 4 demonstrate that the low-frequency part of voltage pulse spectrum is close to a rectangular pulse. The lack of low-frequency oscillations with frequency *f*_1_ is explained by the low efficiency of excitation of the flexural acoustic oscillations in a symmetric structure. As seen in Figure 4(c) and in the frequency spectrum of Figure 4(d) the high-frequency modulation of the generated pulse corresponding to the excitation of planar acoustic oscillations with frequency *f*_2_ ≈ 230 kHz appeared only at large *Ĥ* ≥ 17 kOe.

### 3.2. Discussion and Numerical Estimates

Figure 3(a,c,e) and Figure 4(a,c) (right axis) also present the calculated time dependence *λ*(*H*(*t*)) × *λ*_s_^−1^ where *λ* = *λ*_11_ + *λ*_12_ is the magnetostriction coefficient, *λ*_s_ = *λ*_S,11_ + *λ*_S,12_ is the saturation magnetostriction and *λ*_11_, *λ*_12_ are the longitudinal and transversal magnetostriction coefficients, respectively. The dependencies *λ*_11_(*H*) and *λ*_12_(*H*) were measured by the strain-gauge method [17]. It is seen that the running average of *u*(*t*) follows the time variation of *λ*(*H*(*t*)) × *λ*_s_^−1^ very well. In the quasi-static regime the voltage generated by the layered structure depicted in Figure 1 is proportional to the magnetostriction: *u*(*t*) = *u*_omax_ × *λ*(*H*(*t*)) × *λ*_s_^−1^, with *u*_omax_ depending on the material parameters and the geometry of the sample. From Figures 3 and 4 one gets *u*_0max_ ≈ 4.8 V and 24 V for bi- and symmetric trilayer structures, respectively. If the magnetostriction is driven into saturation an almost rectangular pulse *u*(*t*) is generated. An observed slow decay of *u*(*t*) at the pulse top in Figure 4(a) can be attributed to the electrical relaxation processes in the PE layer [18,19].

The maximum value *u*_0max_ of *u*(*t*) for the symmetric trilayer structure is almost five times larger than that for the bilayer structure. It is achieved when the magnetostriction of the FM material is saturated. Assuming homogeneity of the internal magnetic field in the FM layer and uniform stress over the thickness and plane of the structure, the following expression can be obtained for a symmetric trilayer structure in the low-frequency range [20,21]:

(1) u0max=d31⋅(1−v)⋅v⋅λS⋅(ap+2am)2⋅d312⋅(1−v)−(s11p+s12p)⋅ɛ33⋅(1−v)−(s11m+s12m)⋅ɛ33⋅v,

where *d*_31_ is the piezoelectric coefficient; *v* = *a*_p_/(*a*_p_+ 2*a*_m_) is the volume fraction of PE phase; *λ*_S_ is the saturation magnetostriction with *λ*_S_ = *λ*_S,11_ + *λ*_S,12_; ^p^*s*_ij_ is a compliance coefficient of the PE phase; ^m^*s*_ij_ is a compliance coefficient of the FM phase; *ε*_33_ is the effective permittivity of the PE phase. Parameters of CoFe and PE layers are given in Table 1. *ε*_0_ = 8.85 pF·m^−1^ is the dielectric permittivity of vacuum.

Substituting the known parameters of FM and PE layers into Equation (1) *u*_0max_ = 33 V is obtained. Despite of the approximations there is good agreement with the experimental results. Note that a one-dimensional theory [16,22] underestimates *u*_0max_ (*u*_0max_ = 17.9 V). Equation (1) cannot be directly applied to a bilayer structure due to the bending of the bilayer sample. The much lower value of *u*_0max_ for a bilayer structure can be qualitatively explained in the following way. First, the amplitude is decreased, since the thickness of the FM material is halved. Formal substitution in Equation (1) gives a 20% decrease in amplitude. Hence, the voltage is decreased mainly as a result of bending of the sample. It is known that in pure bending the mechanical strains vary linearly over the thickness of the structure: there is compression at one surface and expansion at the other surface, the plane with zero strain (the middle plane) lies inside the sample. If the thickness and compliance of the FM and PE layers were identical, then this plane would be held exactly in the middle of the structure. Then the electric field across the thickness of PE layer would change linearly, with maximum at the free surface and zero at the inner surface. In this case (because of the linearity of the field distribution) the voltage between the electrodes would be further diminished by a factor of 2. Due to the bending the middle plane is actually located inside the PE layer at a distance of ≈ 37.4 μm from the bilayer interface [23]. The electric field changes its sign inside the PE layer, and (after integration over the thickness) there will be even less voltage between the electrodes.

The oscillations of *u*(*t*) cannot be explained by the quasi-static theory. The observed oscillation frequencies are much larger than *τ*^−1^. To the best of our knowledge, in the absence of bias field and with long excitation pulses such oscillations are observed experimentally for the first time. We are aware of only one paper [24] reporting a similar phenomenon as a result of numerical modeling.

The oscillations of *u*(*t*) could only be detected if the amplitude *Ĥ* exceeds a certain threshold value (compare right columns in Figures 3 and 4). The oscillations of *u*(*t*) start practically from the beginning of *H*(*t*) and not after *λ*(*t*) reaches saturation. The second impetus to these oscillations is caused by the trailing edge of *H*(*t*).

**Figure 5.**
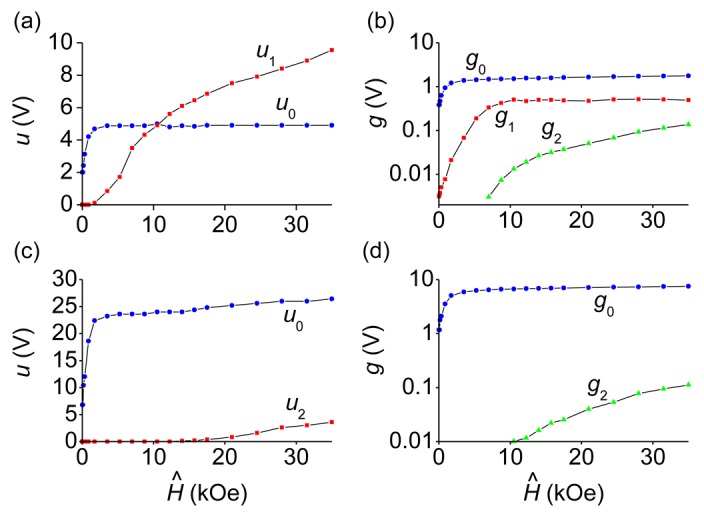
Dependencies of the characteristics of the generated pulse (*u*_0_, *u*_1_, *u*_2_) and the characteristics of its spectrum (*g*_0_, *g*_1_, *g*_2_) for the CoFe/PZT-structure ((**a**) and (**b**)) and the CoFe/PZT/CoFe structure ((**c**) and (**d**)) on the amplitude of the pulse magnetic field *Ĥ*. The lines connecting experimental points serve as a guide to the eye.

Figure 5 shows the dependencies quantitatively characterizing the response of planar bi- and trilayer FM-PE structures on the magnetic pulse with the increasing amplitude *Ĥ*. These dependencies are plotted using data similar to those presented in Figures 3 and 4. It can be seen that for the CoFe/PZT structure the amplitude of the generated pulse initially rises sharply with the increasing *Ĥ* and then reaches saturation *u*_0_ ≈ 4.8 V at a field strength *Ĥ* ≈ 1.5 kOe. The clipping of the pulse amplitude is attributed to the saturation of magnetostriction in the CoFe layer, which occurs just at field strengths of $H_{\text{S}}^{\text{Co}}\approx 1.5$ kOe. At the same time, the amplitude of the low-frequency oscillation *u*_1_ and the amplitude of the corresponding harmonic *g*_1_ in the signal spectrum are increasing. At a field strength of *Ĥ* ≈ 6 kOe the amplitudes *u*_1_ and *g*_1_ reach saturation. At the same field strength the high-frequency oscillations at the frequency *f*_2_ with the amplitude *u*_2_ and the amplitude *g*_2_ of the corresponding harmonic in the spectrum continue to monotonically increase with growing *Ĥ*. For the CoFe/PZT/CoFe structure the amplitude of the generated pulse initially increases with the rising *Ĥ* and then is limited at *u*_0_ ≈ 24 V when *Ĥ*_S_ ≈ 3.5 kOe. The increase of the limiting field of the pulse amplitude, as compared with two-layer structure, is due to the increment in the total thickness of the ferromagnet, resulting in an increase of the demagnetization field [25]. When the field pulse amplitude reaches *Ĥ* ≈ 17 kOe, high-frequency oscillations appear at the top of the electrical pulse. The amplitude *u*_2_ of these oscillations and the harmonic amplitude *g*_2_ in the spectrum continue to grow with increasing amplitude of the field pulse.

The growth of the oscillation amplitudes *u*_1_ and *u*_2_ with the increasing excitation pulse magnitude *Ĥ*, even after the pulse amplitude *u*_0_ was saturated (see Figure 5), can be explained as follows. For the magnetic pulse of fixed duration, the increase of the magnitude *Ĥ* results in the corresponding growth of the magnitudes of harmonics with frequencies *f*_1_ and *f*_2_ in its frequency spectrum. The higher are the magnitudes of these harmonics, the more efficient is the excitation of bending and planar acoustical oscillations of the sample leading to the higher magnitudes of the generated AC voltages. Because of the resonance enhancement of deformations in the PZT layer, the amplitude of AC ME voltage can exceed the voltage *u*_0_ resulting from the static deformation, as observed in experiment (see Figure 5(a)).

To explain the origin of the modulation of generated voltage pulses, let us estimate the frequency of acoustic oscillations in the CoFe/PZT bilayer structure. The eigenfrequencies of the lowest mode for flexural (*f*_1_) and planar (*f*_2_) oscillations of the free plate are given by:

(2) $$f_{1}=\frac{z}{L^{2}}\sqrt{\frac{J}{s\rho A(1-\mu^{2})}}\text{and}\;f_{2}=\frac{1}{2L}\sqrt{\frac{1}{s\rho (1-\mu^{2})}},$$

respectively. Here *z* = 3.56 is a coefficient, *μ* is the Poisson's ratio, *s* is the compliance, *J* is the cross-sectional moment of inertia, *A* is the cross-sectional area of the plate and *ρ* is the density. For a bilayer structure with non-uniform thickness, one should take *A* = *b*(*a*_p_ + *a*_m_), *J* = *b*(*a*_p_ + *a*_m_)^3^/12 and *ρ* = (*ρ*^p^·*a*_p_ + *ρ*^m^·*a*_m_)/(*a*_p_ + *a*_m_), where *ρ*^m^ and *ρ*^p^ are the densities of FM and PE layers, respectively. The effective value of compliance *s* can be derived by averaging either the quantity itself or the Young's modulus *Y* = *s*^−1^: *s* = (^p^*s*_11_ × *a*_p_ + ^m^*s*_11_ × *a*_m_)/(*a*_p_ + *a*_m_) or *Y* = (^p^*s*_11_^−1^ × *a*_p_ + ^m^*s*_11_^−1^ × *a*_m_)/(*a*_p_ + *a*_m_). Remarkably, substitution of the effective value for either *s* or *Y* into Equation (2) gave close upper and lower estimates of the experimentally determined values of *f*_1_, *f*_2_: 32.3 kHz < *f*_1_ < 37.1 kHz, 185 kHz < *f*_2_ < 212 kHz (*μ* = 0.35 was used). This confirms the excitation of bending and planar acoustic oscillations in the structure. The appearance of the oscillations of the generated voltage due to the excitation of acoustic oscillations in the structure may lead to limitation of the dynamic range of ME sensors. Since the frequencies of acoustic oscillations are inversely proportional to the dimensions of the sample, at given magnetic-pulse length unwanted oscillations of ME voltage may be avoided by using smaller structures.

The quality factor of bending (*Q*_1_) and planar (*Q*_2_) acoustic oscillations excited in the structure can be found using the exponential decay rate of the generated alternating oscillation voltage amplitude in Figure 3(e) and Figure 4(c). The estimates yield *Q*_1_^Co^ ≈ 40 (56) and *Q*_2_^Co^ ≈ 78 (90), respectively. Here and in the following the numerical values of the quality factor given in brackets denote those obtained by the HFM method.

Similar measurements were made for bi and trilayer structures (Ni/PZT and Ni/PZT/Ni) containing layers of ferromagnetic nickel. In this case the clipping of the generated voltage pulse (*u*_0max_ = 1.9 V, 4 V for bi- and trilayer structures, respectively), the appearance of oscillations with the voltage amplitude *u*_1_ and the harmonic frequency *f*_1_ ≈ 38 kHz in the spectrum for the asymmetric (Ni/PZT) structure and the appearance of oscillations with the voltage amplitude *u*_2_ and the harmonic frequency *f*_2_ ≈ 217 kHz in the spectrum for the symmetric (Ni/PZT/Ni) structure have also been observed with the growing amplitude *Ĥ* of the magnetic-field pulse. However, since the saturation field of magnetostriction for the Ni layer *H_S_*^Ni^ ≈ 0.5 kOe is smaller than that for the layer of CoFe all the effects have been observed at lower magnetic fields. The quality factors of bending and planar acoustic oscillations in structures with Ni layers are *Q*_1_^Ni^ ≈ 65 (60) and *Q*_2_^Ni^ ≈ 80 (75), respectively. In all investigated samples the quality factors of electromechanical resonances had similar values for the both types of excitation (magnetic pulse or HFM).

### 3.3. Efficiency of ME Interactions

**Figure 6.**
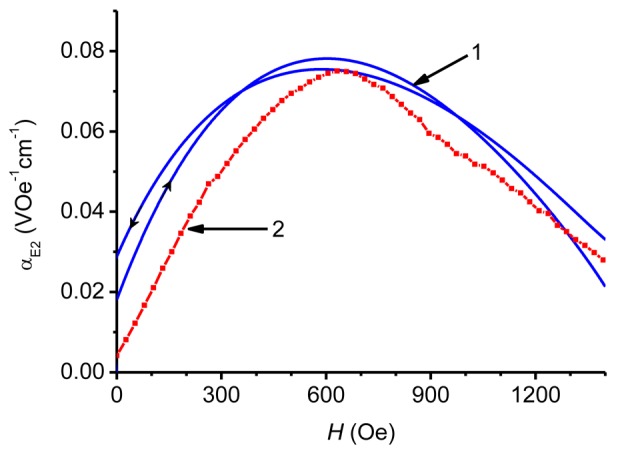
Dependence of the ME interaction efficiency *α*_E2_ of the field strength *H* for the CoFe/PZT structure obtained by the pulse method (1) and by the HFM method at *f* = 1 kHz (2). The arrows indicate the variation of magnetic field strength *H* with time. The line connecting the experimental points in curve 2 serves as a guide to the eye.

**Figure 7.**
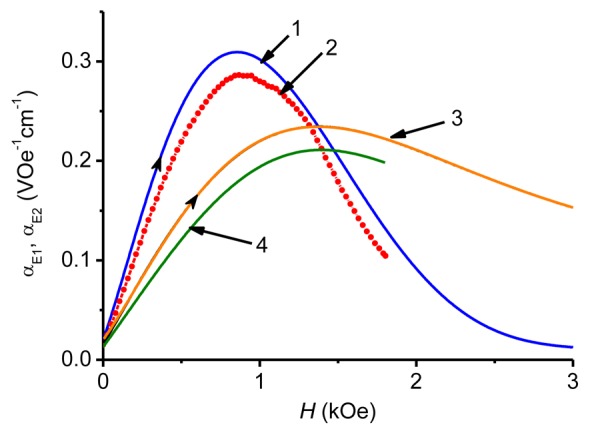
Dependence of the ME coefficients *α*_E2_ (1, 2), *α*_E1_ (3, 4) on the magnetic field strength *H* for the CoFe/PZT/CoFe structure obtained by the pulse method (1, 3) and by the HFM method at *f* = 1 kHz (2, 4). Curves 2 and 3 are measured directly. Curve 1 is calculated from function 3 using the procedure described in the text. Curve 4 is obtained by integrating the function 2 according to Equation (5). The arrows indicate the variation of magnetic field strength *H* with time. The line connecting the experimental points in curve 2 serves as a guide to the eye. For curves 2 and 4, the range *H* < 1.8 kOe is limited by the experimental configuration.

Finally, how the results of pulse measurements can be used to rapidly characterize a ME material must be demonstrated. The measured output voltage *u*(*t*) can be directly recalculated into the nonlinear ME coefficient:

(3) $$\alpha_{E1}=\frac{u(t)}{a\cdot H(t)},$$

where *a* is the total thickness of the sample. Alternatively, the low-frequency (*f* ∼ 10^−2^–10^3^ Hz) ME voltage coefficient for the HFM (linear effect):

(4) $$\alpha_{E2}=\frac{\delta u}{a\cdot \delta h},$$

is often used in applications. In Equation (4) it is implied that an external field *H(t)* = *H* + *δh* × sin(2π*ft*). To find the field characteristics *α*_E2_(*H*), one should use the measured dependences of the field pulse *H*(*t*) and voltage *u*(*t*), find numerically their derivatives *δH*(*t*)/*δt* and *δu*(*t*)/*δt*, calculate *α*_E2_ = (1/*a*) × (*δu/δH*) at each time point and then plot the dependence of this quantity as a function of the field strength *H* at given time *t* [12]. The range of the field characteristics *α*_E2_(*H*) is determined by the amplitude *Ĥ* of the field pulse. To improve the accuracy of the calculations, it is advisable to choose the voltage pulses without undulation corresponding to the excitation of acoustic oscillations in the structure. As an example, Figures 6 and 7 show the field dependence *α*_E2_(*H*) for the bi- (CoFe/PZT) and symmetric trilayer (CoFe/PZT/CoFe) structure found by the described method when the structures were excited by field pulses with a duration of 450 μs and an amplitude of 4.5 kOe. Before numerical differentiation the *u*(*t*)-data were processed by a running average filter and spline interpolation. For comparison, Figures 6 and 7 present the function *α*_E2_(*H*) measured by the HFM method at *f* = 1 kHz. A very good agreement between the results obtained by the pulse method and the HFM method is seen. For the bilayer CoFe/PZT structure, the maximum of *α*_E2_(*H*) is observed at *H* = *H*_m_ = 650 Oe, what is in agreement with previous observations [16]. The hysteresis in the dependence α_E2_(*H*) obtained by the pulse method can be clearly seen. It is attributed to the hysteresis behavior of both FM (magnetostriction *versus* magnetic field) and PE (polarization *versus* electric field) materials [26]. To avoid inaccuracy connected with this hysteresis, the ascending part of the magnetic pulse should be employed for characterization, as illustrated in Figure 7. For a symmetric trilayer structure (Figure 7), the maximum of *α*_E2_(*H*) shifts to the larger values of *H* (*H*_m_ ≈ 850 Oe), which is explained by the larger demagnetizing factor due to the two FM layers [25]. Note that both ME coefficients *α*_E1_(*H*) and *α*_E2_(*H*) are connected by a simple relationship:

(5) $$\alpha_{E1}=\frac{1}{H}\cdot \int_{0}^{H}\alpha_{E2}(H^{\prime })\cdot dH\prime .$$

**Figure 8.**
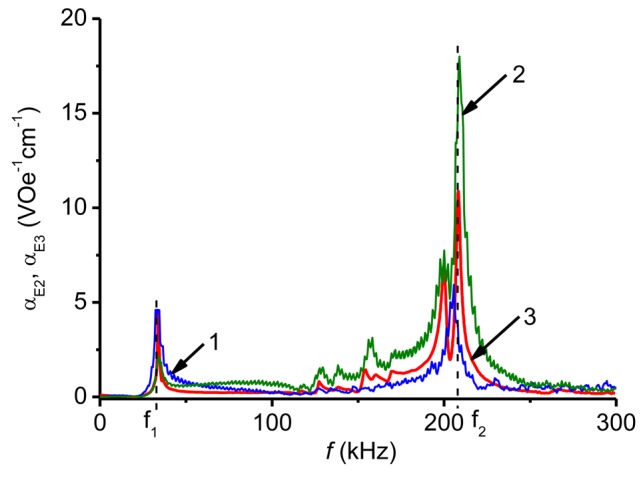
Dependence of the ME coefficients *α*_E2_, *α*_E3_ on frequency *f* for the bilayer CoFe/PZT structure measured by the pulse method (1,2) and by the HFM method (3). Curve 1 is obtained at *Ĥ* = 11.8 kOe and curve 2 at *Ĥ* = 38 kOe.

Indeed, the coefficient *α*_E1_(*H*) obtained from Equation (5) by the HFM method (Figure 7, curve 4) is in very good agreement with that measured by the pulse method (curve 3). For many applications, the frequency response *α*_E2_(*f*) is also of a great interest. Here Equation (4) also applies and the characteristics *α*_E2_(*f*) are usually measured at such a bias field *H*_m_, where the piezomagnetic coefficient *q*(*H*) has its maximum value. At the given excitation frequency *f* the maximum of *α*_E2_(*H*) is observed at *H* = *H*_m_. Figure 8 shows the dependence *α*_E2_(*f*) obtained with a bilayer CoFe/PZT structure by the HFM method at a bias field strength *H*_m_ = 650 Oe. The definition of *α*_E2_(*f*) used in the HFM method cannot be directly transferred to the pulse method, since there is no magnetic bias field applied. In [12] the following characterization methodology was proposed: use the measured time dependences of the field pulse *H*(*t*) and the voltage pulse *u*(*t*), find the frequency spectra of pulses *h*(*f*) and *g*(*f*) by means of the Fourier transform [27] and then calculate the frequency response α_E3_(*f*) from formula:

(6) $$\alpha_{E3}(f)=\frac{g(f)}{a\cdot h(f)}.$$

Figure 8 also displays the dependences *α*_E3_(*f*) for the CoFe/PZT bilayer structure found by the described method when the structure is excited by field pulses with a duration of 450 μs and an amplitude of 11.8 kOe (curve 1) and 38 Oe (curve 2), respectively. The values for the ME coefficient determined by the two different methods show a semi-quantiative agreement. The local maxima of ME efficiency coefficients *α*_E2_(*f*), *α*_E3_(*f*) at resonance frequencies *f*_1_ and *f*_2_ are pronounced. Interestingly, the fine structure in the frequency dependence of ME efficiency is also largely preserved. As can be concluded from Figure 5 the maxima of *α*_E3_(*f*_1_) and *α*_E3_(*f*_2_) are observed at different amplitudes *Ĥ* of the exciting pulse (*Ĥ*_1_ ≈ 11.8 kOe and *Ĥ*_2_ ≈ 38 Oe, correspondingly). This maximum value of the ME coefficient is comparable with that achieved by the method of harmonic field modulation: *α*_E3_(*f*_1_, *Ĥ*_1_) ≈ *α*_E2_(*f*_1_, *H*_m_), *α*_E3_(*f*_2_, *Ĥ*_2_) ≈ 1.5 × *α*_E2_(*f*_1_, *H*_m_) (Figure 8). However, the value of *α*_E3_ at another resonance frequency is smaller than the ME voltage coefficient achieved by the HFM method: *α*_E3_(*f*_2_, *Ĥ*_1_) ≈ 0.5 × *α*_E2_(*f*_2_, *H*_m_), *α*_E3_(*f*_1_, *Ĥ*_2_) ≈ 0.5 × *α*_E2_(*f*_1_, *H*_m_) (Figure 8). The results for the trilayer structure are similar to those shown in Figure 8 except for the absence of the resonance peak corresponding to the excitation of bending oscillations (*f*_1_).

## 4. Conclusions

The peculiarities of the ME response of planar composite structures with FM (CoFe alloy or Ni) and PE (PZT) layers on magnetic-field pulses with a length of about 450 μs and amplitudes from 500 Oe to 38 kOe have been investigated.

- It is found that when the amplitude of the field pulse exceeds the saturation field of magnetostriction in the FM layer (more than ≈ 1.5 kOe for the CoFe alloy and more than ≈ 0.5 kOe for Ni) there is a clipping of the amplitude of the generated pulse voltage. The time dependence of the generated voltage follows the time dependence of the magnetostriction in the FM material.
- With further increase in pulse amplitude fields in the bilayer structures, first bending oscillations, leading to low-frequency modulation of the voltage pulse, are excited and then the planar acoustic oscillations, leading to high-frequency modulation of the voltage pulse, appear. In symmetric trilayer structures only high-frequency planar acoustic oscillations are efficiently excited with large amplitudes of the magnetic field pulse.
- It is shown that the data of pulse measurements enables one to quickly find the frequency and field dependences of the efficiency of direct ME interaction in composite structures. Note that these dependences are obtained from a single measurement as compared to two separate measurements required in the HFM method.
- The results obtained can be useful for developing ME sensors of pulsed magnetic fields. To extend the working field range of these sensors FM layers with high magnetostriction saturation field strength should be selected. The appearance of the oscillations of the generated voltage due to the excitation of acoustic oscillations in the structure may lead to limitations in dynamic range of such sensors.

## Figures and Tables

**Table 1.** Material parameters.

**Material**	***s*_11_ (10^−12^ m^2^·N^−1^)**	***s*_12_ (10^−12^ m^2^·N^−1^)**	***d*_31_ (10^−12^ m·V^−1^)**	***ρ* (10^3^ kg·m^−3^)**	***λ*_s_ (10^−6^)**	***ε*_33_/*ε*_0_**
PZT	14.20	−3.7	−315	8.1	-	4,500
CoFe	4.76	−1.66	-	8.12	60	-

## Appendix

**Table A1.** Notation.

**Symbol**	**Meaning**	**Unit**
*A*	Cross sectional area of the sample	mm^2^
*a*	Total thickness of the sample	mm
*a*_m_	Thickness of the FM layer	mm
*a*_p_	Thickness of the PE layer	mm
*b*	Sample width	mm
*d*, *d*_ij_	Piezoelectric coefficient	V^−1^·m
*f_1_*	Frequency of bending oscillations	Hz
*f_2_*	Frequency of planar oscillations	Hz
*f*_S_	Sampling frequency for discrete Fourier transform	Hz
$g(f)=\frac{1}{N}\left|\sum_{n=0}^{N-1}u_{n}e^{\frac{2\pi jnk}{N}}\right|$	Amplitude spectrum of the voltage pulse, *f* = *k* × *f*_S_ × *N*^−1^ [27]	V
*g*_0_= *g*(0)		V
*g*_1_= *g*(*f*_1_)		V
*g*_2_= *g*(*f*_2_)		V
$g_{\text{rec}}(f)=\left|\int_{0}^{\tau }1\cdot e^{j2\pi ft}dt\right|$	Amplitude spectrum of a rectangular pulse	Vs
$g_{\sin}(f)=\left|\int_{0}^{\tau }\sin\left(\frac{\pi t}{\tau }\right)\cdot e^{j2\pi ft}dt\right|$	Amplitude spectrum of a half-sine pulse	Vs
$H(t)=\frac{-4}{3\pi \mu_{0}\cdot {\left(11mm\right)}^{2}}\int u_{i}(t)dt$	Magnetic field strength	Oe
*H*_m_	Magnetic field strength where *q* is maximum	Oe
*H*_S_	Saturation field of magnetostriction	Oe
*Ĥ*	Amplitude of the magnetic pulse	Oe
$h(f)=\frac{1}{N}\left|\sum_{n=0}^{N-1}h_{n}e^{\frac{2\pi \text{jnk}}{N}}\right|$	Amplitude spectrum of the magnetic pulse, *f* = *k* × *f*_S_ × *N*^−1^ [27]	Oe
*I*	Electric current	A
*J*	Cross sectional moment of inertia	m^4^
*j*	*j*^2^ = −1	-
*L*	Sample length	mm
*N*	Number of measurement points for Fourier transform	-
*q*	Piezomagnetic coefficient	Oe^−1^
*Q*_1_	Quality factor at *f*_1_	-
*Q*_2_	Quality factor at *f*_2_	-
^m^*s*_ij_	Compliance coefficient of the FM layer	m^2^·N^−1^
^p^*s*_ij_	Compliance coefficient of the PM layer	m^2^·N^−1^
*u*	Generated ME voltage (see Figure 2)	V
*u*_0_	Amplitude of voltage pulse without oscillations (see Figure 3)	V
*u*_1_	Amplitude of voltage pulse at *f*_1_ (see Figure 3)	V
*u*_2_	Amplitude of voltage pulse at *f*_2_ (see Figure 4)	V
*u*_i_	Induced voltage in the pick-up coil (see Figure 2)	V
*v*	Volume fraction of the PE phase	-
*Y*	Young's modulus	N·m^−2^
*α*_E1_	Nonlinear ME coefficient	V·Oe^−1^·cm^−1^
*α*_E2_	Linear ME coefficient	V·Oe^−1^·cm^−1^
*α*_E3_	ME coefficient according to [12]	V·Oe^−1^·cm^−1^
*ε*_ij_	Effective permittivity	A·V^−1^·m^−1^·s
*λ*	Magnetostriction coefficient	-
*λ*_S_	Saturation magnetostriction	-
*ρ*^m^	Density of FM layer	kg·m^−3^
*ρ*^p^	Density of PE layer	kg·m^−3^
*τ*	Pulse duration	s
*μ*	Poisson's ratio	-
*μ*_0_	Vacuum permeability	H·m^−1^
